# Recovery Attitudes and Recovery Practices Have an Impact on Psychosocial Outreach Interventions in Community Mental Health Care

**DOI:** 10.3389/fpsyt.2019.00560

**Published:** 2019-08-13

**Authors:** Emmanuelle Khoury

**Affiliations:** École de travail social, Université de Sherbrooke, Sherbrooke, QC, Canada

**Keywords:** recovery, community mental health, attitudes, co-construction, ethnography

## Abstract

The most recent mental health policies implemented in the province of Québec, Canada, have emphasized recovery-oriented mental health practice. Part of this impetus has resulted in significant importance placed on the development of community mental health models in the public health system. The forms of community mental health programs have evolved considerably over time in Québec but are largely inspired by the evidence-based model of Assertive Community Treatment (ACT). However, if mental health policies and programs in Québec are now emphasizing the role of community mental health, it is also clear that actors on the field are implementing the evolving practice paradigms that dominate our mental health policies, such as recovery, participation, citizenship, in a variable way (1, 2). This article presents an ethnographic inspired research study conducted in 2014 and aims to contribute to the understanding of how recovery-oriented mental health policies are understood and implemented in an ACT team in downtown Montréal, Québec. With the aim of developing integrated knowledge on the issue of recovery in mental health and the conditions it presupposes, this research draws on field experiences from various actors, including service users with severe mental health problems, typically with concomitant disorders and complicated by substance use and/or living in a situation of homelessness. Using a critical constructivist approach, the research sought to a) explore how participants (stakeholders, users, and psychiatrists) achieve their social order; b) understand the meaning of recovery in mental health for participants and the actions associated with recovery as a process or as a practice; c) apprehend the potential of community interventions to connect the individual to the collective. The results indicate that the (over)use of medicolegal tools and the unchanging conception of “madness” represent obstacles to the sustained development of interventions centered on the person, his living conditions, and his recovery. Nevertheless, many interactions between service providers and service users indicate the potential for emerging recovery-oriented practice interventions, particularly when those interactions are based on positive and egalitarian conceptions between service providers and service users that led to the development of spaces for the co-construction innovative practice approaches.

## Introduction

The concept of mental health recovery is the dominant organizing principle for public mental health services in many countries (1). This has resulted in important changes in the strategic direction and in the practice guidelines of mental health delivery systems. For example, recovery-oriented practice guidelines (2) in Canada stipulate that recovery “occurs in the context of one’s life” (p. 38) and points to the imperative need for mental health professionals to consider and act upon social determinants of health. The literature has supported the idea that, although an individual will go through the process of recovery, professional intervention can facilitate this process (3–6). This requires certain critical values focused on egalitarian and relational attitudes (6), hope, implication, and relationships (7) that are supported by both training and organizational structures (1). The integration of mental health recovery as an approach and philosophy underlying mental health policy at an international level underscores the importance of the recovery concept; it is process oriented, person focused, and shapes governance structures toward social inclusion, quality of life, citizenship, and participation. However, there are documented difficulties in implementing a recovery-oriented practice approach (1, 8–11) that has led to misapplications, misunderstandings, and critiques (12, 13).

In Québec, mental health policies and care (Mental Health Action Plan 2005–2010; 2015–2020, hereafter MHAP) have evolved with an impetus to develop recovery-oriented community mental health teams, such as Assertive Community Treatment (ACT). Part of the continuum of mental health reform, community mental health care offers the possibility to avoid potentially stigmatizing psychiatric care by placing the person and her living environment at the center of care services. In Québec, ACT teams were developed with this care logic but also with a logic of cost efficiency (14–16).

The ACT program is a model that was developed within the walls of a psychiatric institution as an alternative to hospitalization in the context of deinstitutionalization; it is defined as providing round-the-clock, individualized psychiatric services in a person’s home or community (17). However, it has a history of coercive and intrusive interventions that are almost singularly focused on pharmacological adherence to manage symptoms (18, 19). The objective of ACT is to provide comprehensive outreach in the community where service users can access the same type of treatment team they would access if they were in an inpatient setting. Elaborated in the Québec landscape, the ACT program aims to help people with serious mental health problems, often with substance use disorders or concomitant disorders, develop their individual competencies. The objective is to promote their autonomy and social integration by shifting (20) psychiatric treatment and psychosocial follow-up out of the hospital and into the individual’s community [Gélinas, 1997, in Ref. (18)], particularly through supportive housing and targeted work placement programs.

The aim of this study was to shed light on the interactional experiences of providing and using services from an ACT program, particularly in an urban setting, wherein service users presented with complex mental health and social problems, including dual diagnosis for substance use and concomitant disorders. In Québec, the Centre for Excellence in Mental Health regulates and evaluates the ACT teams.^1^ However, until now, there has been very little knowledge regarding the daily interactions, actions, and relational dynamics that support recovery within an ACT team, despite findings that point to the importance of social processes and social dynamics in reinforcing a recovery culture (1, 21). Based on these previous findings, the hypothesis was that the relationship between service users and service providers would be primordial to reports of subjective recovery journeys, and that service providers would capitalize on the proximity of their interactions to surmount organizational constraints when intervening with complex issues.

I conducted a critical ethnography at an urban ACT team whose service users experienced serious mental health problems combined with complex social problems, such as poverty, isolation, and gentrification. Many service users also had concomitant addiction disorders, and the professionals on the field had to adapt their interventions and recovery perspectives *in situ* to respond to the particular needs and aspirations of this population.

## Materials and Methods

### Methodology

Critical ethnographies are a way to provide an informed reflection based on real-world contact with mental health service users and providers in highly marginalized and simultaneously gentrified urban areas for a sustained period. A key strength of ethnographic case studies is the ability to tease out the underlying value systems of the specific organizational cultures and their contexts, which can then provide key lessons to understand other situations (22). Its questioning of the relationship of social order and social structures and its methods of “reconstructing social reality by privileging multiple voices” (23) are techniques indispensable to describing and explaining relationships between people and systems within the larger political, economic, social contexts (24). At the same time, critical ethnography is considered to be a methodology that refuses to separate theory from methods (25), thereby offering a way for professionals to become “more consciously aware of how they take up their professional authority in managed mental health care contexts” (26, p. 173). Moreover, ethnographic inquiry results in not only a “thick description” (27, p. 10) of the culture being studied but also an inductive analytical strategy that requires the researcher to uncover relationships in the context of the observational and interview data. I adopted a cross-paradigm framework, including Healy’s (28) conceptual model of critical practice that speaks to the importance of context and power relations in intervention construction and on Garfinkel’s (29) ethnomethodological focus on the interactional and *in situ* nature of interventions. The use of multiple frameworks is coherent with qualitative research and enhanced the research to “to see in new and different ways what seems to be ordinary and familiar” (30).

The ethical considerations specific to ethnography were considered, and they were attended to through a consistent reflexive stance and structured journaling. Although Husserl’s notion of “bracketing” and putting aside historical and cultural assumptions to attain objectivity is vigorously contested (31), the reflexive ethnographic researcher can acknowledge these assumptions to be more thoughtful, critical, responsible, and informed of potential biases, expectations, and judgments.

### Sampling and Data Collection

I was on site for 3 days a week over the course of 7 months engaging in participant observation. The interview component consisted of six interviews with service users and 12 interviews with health and social care professionals, including two psychiatrists, three nurses, four social workers, two psychoeducators, and one criminologist with years of experience ranging from 6 months to 10+ years. The service users who participated in the critical ethnographic study had been involved with the program from 1 month to 5 years. Although only six service users were interviewed, the care trajectories of approximately 20 service users were followed in observation and through access to their case files. During the study period, I observed service providers accompany two service users to a long-term addiction rehabilitation facility; one service user was hospitalized against his will after a substance-induced psychotic episode; one service user’s intervention plan focused around his debt accumulation for the purchase of illegal substances; and another was concurrently followed at the methadone clinic. I used a strategy of triangulation^2^ of sources by using three data collection techniques—participant observation, document analysis and case file analysis, and individual interviews.

### Measures and Analysis

Consistent with a qualitative tradition, the data were analyzed using the NVIVO software and by using techniques of thematic analysis that began with an initial open coding phase. Understanding the concept of recovery from the subjective perspective of participants was achieved not only through inductive coding but also through identification of i) the influence of agency and policy contexts on practice, ii) the analysis of practice descriptions, and iii) recovery-oriented perspectives of the implementation of the mental health policy. The analysis considered microlevel dimensions of recovery, such as social interactions with friends, family, and neighbors, mesolevel dimensions, such as social interactions with professionals and institutions, and macrolevel dimensions, such as community engagement and participation. The ethnographic component of the 2014 study meant that data collection and analysis were simultaneous. Analysis was very tangled up with every stage of the research process (22).

Funded by the *Fond de recherché du Québec-Société et culture* (FQRSC) doctoral award, this ethnographic study is governed by deadlines respecting ethics and integrity. The study received approval from the Université de Montréal’s Research Ethics Board and Université de Montréal Health Centre’s Research Ethics Board. All of the participants signed consent forms and kept a copy of the signed agreement. The informed and free consent of participants was assured.

## Results

The everyday world of this urban ACT team is dynamic and in action. My findings related to the subjective participant meanings and accomplishment of recovery-oriented mental health care that are embedded in this dynamic active state.

The thematic analysis underscored three key elements to the interactions and actions that are derived from the social processes and organizational structures of this ACT team. These three elements, flexibility in practice, complexity of practice, and relationships in practice, are located at the intersection of difficult practice moments, which I refer to as “practice tensions.” These three elements sum up the particular nature of the culture of intervention of an urban ACT team with a population experiencing complex mental health problems and social problems as well as the meanings and actions involved in recovery-oriented attitudes and practices.

### Flexibility in Practice—A Key Component of Recovery-Oriented Practice

Major differentiating factors of the ACT team compared to other specialized mental health care teams are the flexibility and intensity that are hallmarks of the ACT fidelity scales. What do these descriptors look like in real life? The organizational structure of ACT provides a good foundation for service providers to be flexible with their timing and schedule and for all service providers to influence the overarching team perspective on care. That means that when they visit a service user for a coffee, to deliver or administer medication, or for a visit, they can take as long as the service user needs. Sometimes this is 5 min, and sometimes a simple medication delivery becomes a 30-min intervention. The ACT program’s continuity of service through its connection to a parent institution is a key factor ensuring flexibility and clinical autonomy, as per one of the psychiatrists who explains:

*It’s clearer and clearer in the literature that ACT is a flexible and adaptable platform … because we are attached to a hospital, we can ensure continuity … we have the privilege of direct admission, we don’t need to negotiate…*

Thus, the notion of flexibility remains rooted in a hospital-centric approach where it is understood by the link with admission units and the psychiatric emergency. The traditional case management model does not seem to provide a context for making sense of the construction of interventions that can influence community links, social cohesion, or social participation.

Service providers described tensions in their desire to develop interventions that are recovery oriented and the current organizational framework, which is influenced by a rigid, institutional design. However, the ethos of this team, that is, the way it believes that service users should behave, develop, and feel (27), influences the meaning prescribed to interactions and influences the flexibility that is promulgated regarding both through relationship building and within the organizational framework.

The intimacy that both the intensity and the intrusiveness of ACT programs requires can be potentialized to gain in-depth knowledge and trust with a service user. This is particularly supportive of recovery-oriented interventions with service users who have complex social problems or present with addiction or concomitant disorders. As explained by one service provider:

*That’s the difference, it’s not in an office … being at a client’s home, it’s intimate. The home can tell you a lot about a client. We have access to things that you wouldn’t access in an office meeting.*

Flexibility exists in the professional autonomy experienced by service providers. Although the choice of practice approaches is somewhat regulated by the Centre for Excellence in Mental Health based on fidelity to the TMACT scale (32), many innovative interventions are constructed *in situ* to respond to the diverse and complex needs of service users. These include street-level work, such as meeting a service user at a downtown bus station when she returns from an addiction rehabilitation center or meeting a service user daily in his home to structure his budget and his daily activities to reduce his recourse to debt accumulation and substance use.

For example, this particular ACT team demonstrated its harnessing of the flexibility of the ACT program to develop practices that are not included in their fidelity scales and evidence-based mandate. These attitudes and practices led to the development of new services within the team, such as a mini-team to intervene specifically with a homeless population experiencing mental health problems. This new way of working, above and beyond their mandate of ensuring supportive housing and medication adherence, is an example of flexibility in intervention, wherein recourse to medicolegal tools or pharmacology was not perceived as the only option for service providers.

The personal influence that individuals have on how flexibility is defined should not be underestimated. Regardless of that, there is an important inescapable rigidity in the tools that are available to the team. These are mostly related to the way medication and a reductionist biomedical perspective continue to have a structuring role. In many ways, this is an elite team of mental health professionals who have experience, knowledge, and a significant amount of power as they enter into people’s homes and communities. The notion of flexibility in practice is an element in tension because, despite the community location, there are few community-focused interventions. The choice of intervention is constructed according to the subjective values of the actors in action rather than by the technical platform of the ACT model that is physically located in a community-based setting.

### Complexity of Practice—A Key Component to Addressing Authority and Autonomy in Practice

The complexity of practice is evidenced as a situated action wherein the tensions in the position of ACT as a specialized, elite, and experienced team and the subjective concerns regarding coercion, authority, and risk management are explicated. Situated action is at the heart of an ethnomethodological and interactionist research perspective because it emphasizes how participants use common-sense practices to produce, analyze, and make sense of each other’s actions and circumstances.

This elite team also expressed feelings of powerlessness at being the end of the line of care. Court orders offer an upper hand in negotiations to ensure compliance and treatment. As one social worker explained:

*This is the last stop in services for these people … I think once they are back on their feet, then they are ready … but it’s difficult to establish a relationship, we represent an authority that reminds them of their illness … the people followed here are very unstable … We need court orders.*

The program priorities of both avoiding hospitalization and maintaining autonomous housing as well as the emotional need for service providers to alleviate their feelings of helplessness sometimes led to service providers attending to clinical-administrative priorities rather than person-centered clinical impacts. As one service provider told me, “If the person decompensates, we’ll look like clowns.” The tension for the service users is that they are relieved to be avoiding multiple hospitalizations in collaboration with the ACT team but they also reported experiencing uneasiness with the supervision and control of their actions and interactions.

These statements belie the control and surveillance that service providers feel are necessary to accomplish their roles, manage risk, and interact with service users. One of the service providers, Robert, explained that he justifies the imposition on service users that his role as an ACT service provider requires by framing it as a way to force collaboration. Other service providers echoed the sentiments that “there is no choice with an ACT clientele but to be coercive.” The team lead offers a softer approach to the tension between coercion and collaboration. She explains that the coercive nature of the ACT program, engrained into its very *raison d’être*, requires the service providers to be “strategic in their negotiations [with the service user] so that the outcome is in the service user’s advantage.” This is often accomplished by “striking a deal” with the service user and avoiding legal orders. For example, there was one situation in which there was the possibility for a young adult, who came to the service when she was living on the streets and using heroin, to live in stable institutional housing. Specifically, the team agreed that they could offer this service user less frequent visits and more weekly money to help her feel secure in exchange for her accepting the housing agreement.

The central implication of this element, complexity in practice, is that recovery actions and interactions are dependent on the conception the team has of the service user, the subjective service provider beliefs, and the understanding of risk management that is at the heart of daily decisions of stakeholders. But discursive processes, the team culture of dialogue, and social cohesion among service providers seem to be protective factors in the evolution of risk analysis and subsequent measures put in place. I observed a very cohesive team built by the service providers and the psychiatrists who share their knowledge and concerns informally in the corridors but also officially in daily meetings. Internal decision making is horizontal, and service providers themselves often challenge the dominant medical discourse. They consciously avoid using diagnostic language.

### Relationships in Practice—A Key Component to Building Trust, Hope, and Implication

It is not surprising that service providers’ subjective experiences affect their affiliation and affinity toward certain service users. Therapeutic alliance implies getting to know persons for who they are, their interests, their life stories, and thus going beyond a reductionist description of symptoms. One service provider explained the centrality of the therapeutic alliance:

*A relationship. When we have a relationship with a client, we have everything. The rest is candy. A trusting relationship, a human relationship.*

Service providers and service users made the assertion that focusing on symptoms and medications and even reverting to hospitalizations are “the easy way out.’” The varying complexities of social scenarios remind the team lead, a social worker, of the role of psychosocial elements in the vulnerable and marginal situations that the service users find themselves in. The team lead suggested that her professional standpoint, which is a result of her professional and personal experiences, has made her realize that:

*We need to flirt with risk … I am not afraid of madness and so I have access to madness when I meet with people.*

The relationships, the therapeutic alliances, the community housing, and the social networks that are developed in the ACT structure are not created only within the confines of the four walls of the ACT office. They are also created and developed on the street, in the bus, in cars, in apartments, in short, in circulation in the community. These relationships that are amicable and sometimes even affective are bound by the professional role of the service providers. These are institutional relationships that are uniquely joined at the locus of the human condition, juggling the reality of implicit control through medication and explicit trust building predicated on the acceptance and facility with madness. The affinity among actors is woven by the social links between service providers and between service providers and service users. Although some participants pointed to the long-term follow-up that is accorded within an ACT program as a necessary element to relationship building, this element also exposed tensions regarding sustainable and authentic social integration.

One psychiatrist on the team expressed some distress at having to juggle the biomedical paradigm and more progressive person-centered approaches. However, she suggested that the coercive practices, which are anchored in the traditional psychiatric philosophy, are a status quo that must be worked around rather than revoked:

*How do we deal with the coercive aspect of our job … I find it hard … It is a challenge. I do not want to harm anyone. It is a clientele that is not always easy, the risk is ultimately complex.*

The ACT team, through their frequent contacts with the service users and, for the most part because of the sincere interest from the service providers, has succeeded in initiating a trust relationship with most participants. This also serves to understand, respect, and recognize a person’s fears or concerns. The ACT program activities provided a context in which interventions and interactions among actors take place in intimate settings, such as the service user’s home. The variety of places and spaces for intervention leads to a permutation of many aspects of the lives of the users; I observed that often the service providers’ role paralleled that of friend and family. This leads to a quality of interaction based on special attention to people’s lives and circumstances. Although the intimacy of home visits is often experienced by the service user as an intrusion and management of her private space, it was also welcomed by many and qualified as “human” and “calming.”

The tension lies in the location of these interactions being both a unique opportunity to have access to the singular experiences of the service user but are also obstacles to integration and to actual inclusion in the community. The results demonstrate that the ACT team has become a social network for users. However, clear professional boundaries ensure that the development of this social network is unidirectional, empty of the reciprocity found at the core of human relationships, reducing the interaction to a simple service offer.

## Discussion

In the present research, my aim was to examine both the understandings and affinity to recovery-oriented practice and to understand how and if it is constructed by service users and services providers. The results show that one of the strengths of the constant tug of war the ACT team experiences, whether it be about what they do with the flexibility the program accords them, or how they use tools and professional autonomy to mobilize community resources, or how they respond to social inequalities, is that the dialectic is not suppressed. Although social change is not addressed or mandated, specific microlevel interventions are distinguished based on the service provider’s relationship with the service user and the assessment of a service user’s potential for personal change.

In the ACT team, every action and interaction are parts of a hybrid service culture that is on the one hand person centered and flexible and on the other hand symptom focused and coercive. Once a strong therapeutic alliance has been created in a professional-patient dyad, and in the ACT team it is often on the premise of developing a social relationship, then the line between a paternalistic interaction and one that is egalitarian and potentially collaborative is blurred. The authority inherent in the role of professionals and particularly in their role as professionals in an ACT team is not lost on the service users. One service user, whose relationship with her service providers has been mitigated by their intervention plans that include inpatient addiction rehabilitation services, explains the tensions that she experiences with the professionals who are at once personable and warm and also hold immense power and control over the lives of service users.

The space and place used by the ACT team can have paradoxical impacts on the multiple actors. The occupation of these spaces at different times and in different circumstances gives rise to tensions that are often invisible to the official structure of the program. The social roles that are played by the actors in an ACT team are varied and numerous, echoing the seminal discussions by Goffman (33, 34) that a group, or in this case a team, plays a central role in the actions and interactions of individuals within that team. Once they know the established roles and rules of play, the service providers “improvise” individual actions that are to be chosen based on the effect that they might have on others.

### Proximity, Intensity, and Recovery

The different ways of engaging in relationships in close proximity make up the specificity of ACT interaction and are often referred to by the service providers as “accompaniment.” These actions and interactions are not framed by clinical tools or clinical guides and are often context and person dependent. They can range from feeding a service user’s cat when they are hospitalized to helping them move apartments or to buying groceries and cooking supper together. For service users, the hope and time that are offered through the structure of the ACT team are important for their recovery process specifically as it relates to social relationships.

The service provider-service user relationships, which are embedded in a professionalism that maintains strict boundaries, do not erase power inequalities and the specificity of the belonging to a certain group (service user, professional, psychiatrist). This division is a major challenge for the ACT team as it works toward improving the quality of life, and supporting a life of quality, for service users in the community. Service providers generally concurred that they focus on the observable mental health difficulties, whereas other teams, groups, or services will work in partnership with them to manage and support in other aspects of the person’s life.

### Two-Tiered Recovery Practice

This study found that recovery-oriented practice is accomplished through a form of institutional accompaniment that is developed based on a singular, intimate knowledge of each service user and through a negotiation of outcomes for groups of service users. Service providers believe in the general idea of recovery as per my observations and the interviews, but the construction of recovery-oriented practice is more elusive. There are paradoxes and complexities specifically related to institutional accompaniment. The institution offers a more traditional role of providing a safety net for service users. The discourse of recovery is prevalent among service providers only when asked directly; however, the actions and the sense given to recovery-oriented practice are evidenced through their discursive practices and their innovative and emerging practice. This ACT team seems to accomplish a hybrid type of recovery-oriented practice, in which some service users are externally evaluated as being on a “maintenance” track and others on a “recovery” track. Interventions and relationships are constructed in consequence of the outcome that is a priori determined for the service users. Both tracks include interventions that aspire to positively affect the service user’s social environment (housing, social network, hygiene) and have a symbolic value associated with well-being, recognition, solidarity, and participation. Moreover, the two tracks in this recovery practice are embedded in the role that ACT plays as a proxy, unidirectional social network for most service users. The development of a proxy social network might be stimulated by the social skills training offered by ACT; it might also be reassuring and structuring for service users who require and desire that. However, there is a risk that it becomes a mechanism for “social contention” and limits effective development of sustainable and reciprocal social connections and social cohesion.

Despite the most progressive intentions of service providers, the recovery process and the construction of potential recovery-oriented interventions are negotiated not only for individual service users but also for groups of service users based on social workers’ expectations of that group. Thus, there is a two-tiered approach to recovery for service users evaluated as having a capacity for rehabilitation, who are judged as having adequate insight, and another approach for services users that are judged to have low insight and therefore incapable of rehabilitation for the time being. For the former group, the type of interventions that are constructed can be categorized as “accompaniment” and for the latter group as “maintenance and safety net.”

The two-tiered recovery approach represents a paradoxical institutional arrangement of accompaniment that remains highly individualized and relegates social inequalities to an unexamined background reality. Complex social problems, such as homelessness, are addressed more directly through the development of new organizational structures (PRISM) and approaches (street psychiatry), with the goals of providing solutions to individualized mental illness through medication and housing. Although this outreach is a first step to connecting and building a relationship with certain people experiencing distress, the framework of recovery is not a consideration or used as an orientating approach. Social interventions, both for those service users who are to be maintained in their stability and for those who are grouped into the category as having potential for transformation, are lacking a broader concern for social and collective concerns.

Service providers often cite the organizational constraints and the subsequent legal, medical, and administrative pressures as the most influential factor in the way interventions are conceived and constructed. These constraints and pressures lead to a focus on symptom reduction, harm and risk reduction, avoiding hospitalization, maintenance in the community through housing, and improved social connections/cohesion. The complex social difficulties and inequalities faced by the service users are often through interventions shaped by purpose rather than process and lead to recovery being reconceptualized into an individual responsibility. The context in which that individual must take responsibility for her recovery process, both socioeconomically and clinically, is not a predominant consideration. In other words, a service user, such as Liz, is experiencing feelings of hopelessness, marginalization, and also living with the effects of poverty and stigmatization, within the ACT context, she is able to establish therapeutic relationships that support and accompany her in finding housing, managing her substance use, and connecting with her family. However, Liz does not report being in a process of recovery because her existential goals are not being met. Moreover, the predominant recovery framework that is used is one in which Liz alone is responsible for her recovery, despite the current context in which her treatment, housing, and certain broader life choices are controlled by the very relationship she developed with the ACT team.

### The Importance of Interactions and Relationships

What I have explicated in this research is the appearance of different intervention strategies in ACT mental health practice. Service providers harness their organizational structures and their professional autonomy and knowledge to either a) access privileged moments and spaces for potentially transformative interventions or b) interact with service users through techniques that maintain spatial, temporal, and interpersonal stability. Despite the most progressive intentions of service providers, outcomes are negotiated not only for individual service users but also for groups of service users based on service providers’ expectations of that group. That means that some groups of service users are considered to be on the “recovery track,” and more complex interventions are envisioned, whereas other groups of service users, usually those with more complex problems, are on the “maintenance track,” and more technical interventions are accomplished. The unequal outcomes by level of distress or suffering suggest that stigma or discrimination has become structured into the ACT program and the parent institution, through daily actions and interactions among actors in a mental health team, despite the goodwill and professionalism of many workers. Larger structural inequalities are not only constructed but also maintained within these interactions because service users and service providers are mobilized to accomplish tasks within the public space, outside of the institution, rather than to transform it.

Although service providers might feel empathy, compassion, and even affection for some service users, clear professional boundaries ensure that the development of a social network is unidirectional. Service providers, often social workers, find themselves in privileged spaces to develop relationships and promote dialogue with services users. These spaces can be in cab rides, walking down the street, moving, having a coffee. During these interactions, small acts of kindness, which service providers often dismiss as inconsequential, are interpreted as very meaningful and moving by service users. These small acts of kindness, which are not yet included in best practices literature, are developed when the dyadic relationship enters into a “dialogue” mode. It is perhaps, as suggested by Linda Bourgeois (35), a former service user, the first step to self-transformation and social transformation. These small gestures and acts of kindness are perhaps invisible actions and interactions that serve to reinforce a more flexible and participatory relationship, despite the fact that they are not easily categorized into a specific intervention approach. The quality of the interactions became apparent from how case managers paid attention, listened, and communicated while engaging in these shared activities. This study illustrates that although the structural aspects of SIM provided the context and opportunities for engagement, the quality of the interaction between the case managers and residents played a key role in engagement.

The particularity of the service providers that compose the ACT team is their continued hope, built through professional respect and through an awareness, if not necessarily always the capacity, to intervene in different ways than what is typically sanctioned in psychiatry and in our overarching results-based health and social service sector. The service provider who deals almost exclusively with the Individualised Placement Program explained that his passion for his work is based on his strong belief that it will provide hope for the service users and for himself as a professional.

## Conclusion

In short, in 2014, within this urban ACT team, the question of participative, service user-oriented practices in psychiatry is already being debated. This debate can be understood as situating this particular ACT team as not only a physical space where community mental health work is accomplished but also a social and political space wherein madness is woven into the fabric of the community and of society. This latter occupation unearths many of the constraints and questions regarding the relationship this psychiatric team has with the idea of madness and with the realities experienced by service users. The description of recovery and recovery-oriented perspective within this urban Montreal ACT team unveils an organizational structure that is opening the space for potentially creative and participative actions and interactions among actors—that is to say, interventions that seek out and sustain the participation of service users in their treatment and in their lives in the community. Perhaps paradoxically, this same structure is governed by traditional practices and neoliberal policies that maintain and support traditional professional-patient relationships and cost-efficient treatments. Moreover, the position of this elite team within the psychiatric care structure might even legitimize the use of more coercive practices.

This research offered an opportunity to identify the ways in which service users and service providers understand recovery-oriented practice in action. Specifically, this research demonstrated that the challenges of recovery-oriented practice lie in the conception of the service user in relation to his or her mental health difficulties. The findings indicate that the interactions between service providers and service users, and the subsequent conceptualization of the service user and the interpretation of varying situations, continue to be entrenched in a paternalistic and patriarchal approach. Nevertheless, the study findings show that several service providers offer less medicalized and less paternalistic perspectives than expected. Their interventions seem to be the result of an evolving interpretation, or conceptualization, of “the mental health service user” and of specific situations. It is vital to underscore how representations of a situation or a person can potentially change practice; perhaps the quality of the interpretation of situations and people can actually transform the potential of the human relationship between service providers and service users and practice interventions.

Although the study provides a portrait of a specific community mental health team in an urban environment, the results offer significant practice-near insights and observations into the daily actions and interactions of the team. The literature points to the need for a compounded effort to describe and analyze practice-near understandings of recovery-oriented practice (10) not only to explicate barriers and facilitators of recovery-oriented community mental health practice with service users presenting with complex needs but also to highlight promising and emerging practices.

## Limitations

This study has several limitations. First, ACT teams in Québec are demonstrated to be heterogeneous (15) and adapted to the population they serve. This generalization of the present results across different models could be problematic. As well as the single author observation and analysis of the data. I used the phenomenological approach of “bracketing” (36) and the critical ethnographic stance of ethical responsibility for a researcher’s positionality (31). Like Giorgi (37) in Tufford & Newman (2010), I bracketed by suspending those biases, with the assistance of journal writing, memos, and conversations with my research director to reflect on the forces that have shaped my interpretations during the writing and analysis process. However, I also developed an awareness of my preconceptions before the beginning of the study through reflexive journal writing throughout my doctoral studies. As far as I know, this research was the first that examined the social processes and interactions within an urban ACT team with a critical recovery-oriented perspective. This study aimed to uncover not only the meanings prescribed to recovery-oriented community mental health practice with a population experiencing important mental health difficulties and addiction and concomitant diagnosis but also the actions and interactions that make up practice.

## Ethics Statement

This study was carried out in accordance with the recommendations of the Université de Montréal's Research Ethics Board and the Université de Montréal Health Centre's Research Ethics Board with written informed consent from all participants who kept a copy of their signed agreement. The protocol was approved by the Université de Montréal Health Centre's Research Ethics Board.

## Author Contributions

EK came up with the project idea,in collaboration with her doctoral research supervisor, Dr. Lourdes Rodriguez del Barrio. EK was responsable for the study design, data collection, data analysis, and writing the article.

## Funding

This article was funded by the *Fond de recherché du Québec–Société et culture* (FQRSC) doctoral award. The study was also supported by the Community-University Research Alliance in Mental Health and Citizenship (38).

## Conflict of Interest Statement

The author declares that the research was conducted in the absence of any commercial or financial relationships that could be construed as a potential conflict of interest.

## Footnotes

1 The Centre&amp;rsquo;s Web site specifically notes that the services of these teams are intended to respond to service users with severe mental health problems who may also have concomitant addiction disorders.

2 Guba and Lincoln, 1985;Quivy and Campehoudt, 1995.
